# Supplementary material for: Response of the Endophytic Microbiome in Cotinus coggygria Roots to Verticillium Wilt Infection
Source: J Fungi (Basel). 2024 Nov 15;10(11):792. doi: 10.3390/jof10110792 (PMC11595824; doi:10.3390/jof10110792)
Supplement: Supplementary file 1 [file jof-10-00792-s001.zip › jof-3259377-supplementary.pdf]

Table S1. Alpha diversity of the root endophytic microbiome of Verticillium wilt-affected and healthy *C. coggygria*. RP represents the Verticillium wilt-affected roots; RH represents healthy roots.

Category	Samples	Sobs index	Chao index	Ace index	Shannon index	Invsimpson index
Bacterial community	RP	252.33±31.57a	360.65±83.35a	349.25±130.77a	2.81±0.01a	8.26±0.23a
	RH	914.33±71.02b	1032.70±132.30b	1032.05±116.90b	4.97±0.09b	43.18±12.40b
Fungal community	RP	132.00±3.61a	141.35±6.93a	140.42±6.16a	1.47±0.16a	2.53±0.56a
	RH	34.33±11.02b	38.06±12.99b	38.16±12.99b	1.10±0.38a	2.56±1.04a

Note: Different lowercase letters represent significant difference in the same column ($P < 0.05$)

Table S2. Relative abundance of the dominant taxon in the root endophytic microbiome of Verticillium wilt-affected and the healthy *C. coggygria*.

RP represents the Verticillium wilt-affected roots; RH represents healthy roots.

Taxon		RH	RP	Taxon		RH	RP
Bacterial phylum	Proteobacteria	0.44±0.04	0.62±0.01	Fungi phylum	Ascomycota	0.96±0.05	0.40±0.13
	Actinobacteriota	0.45±0.04	0.35±0.01		Basidiomycota	0.04±0.05	0.60±0.13
	Firmicutes	0.02±0.00	0.02±0.00				
Bacterial class	Alphaproteobacteria	0.31±0.02	0.38±0.00	Fungi class	Agaricomycetes	0.04±0.05	0.60±0.13
	Actinobacteria	0.35±0.04	0.34±0.01		Dothideomycetes	0.94±0.06	0.20±0.11
	Gammaproteobacteria	0.13±0.03	0.23±0.01		Sordariomycetes	0.01±0.01	0.14±0.04
	Thermoleophilia	0.08±0.03	0.01±0.00				
	Bacilli	0.02±0.01	0.02±0.00				

Table S3. Topological properties of the co-occurrence networks in the root endophytic microbiome of Verticillium wilt-affected and healthy *C. coggygria*. RP represents the Verticillium wilt-affected roots; RH represents healthy roots.

Sample numbers		Degree	Degree centrality	Closeness centrality	Network density	Total edges	Positive edges (Percent)	Negative edges (Percent)
Bacterial	RH	15.68±0.47a	0.32±0.01a	0.32±0.01a	0.320	392	205(52.3%)	187(47.7%)
	RP	14.17±3.64b	0.30±0.08a	0.30±0.08a	0.301	340	165(48.5%)	175(51.5%)
Fungi	RH	8.09±4.50a	0.19±0.11a	0.19±0.11a	0.193	174	146(83.9%)	28(16.1%)
	RP	26.37±12.28b	0.55±0.26b	0.55±0.26b	0.549	646	315(48.8%)	331(51.2%)

Note: Different lowercase letters represent significant difference in the same column ($P < 0.05$)

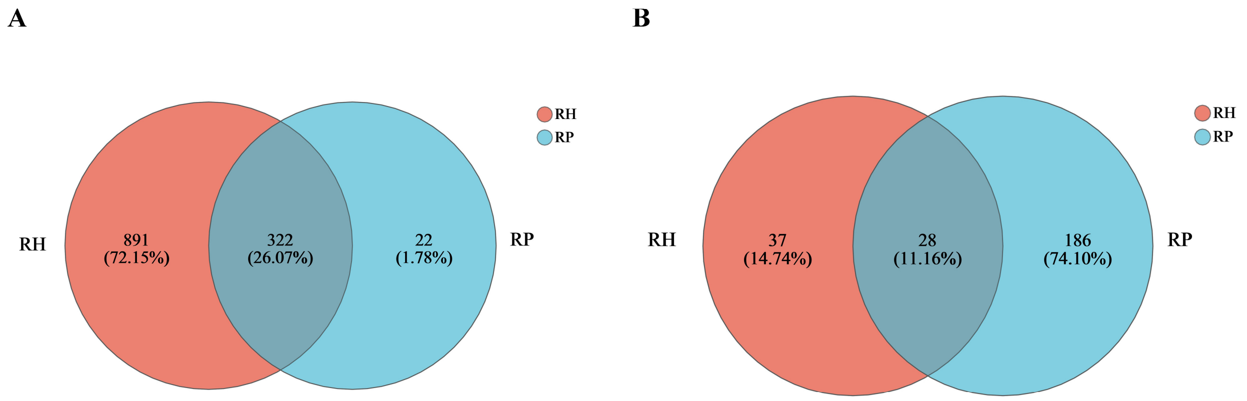

Figure S1. The shared and unique OTUs of the root endophytic microbiome in Verticillium wilt-affected and healthy *C. coggygria*. (A) bacterial community; (B) fungal community. RP represents the Verticillium wilt-affected roots; RH represents healthy roots.

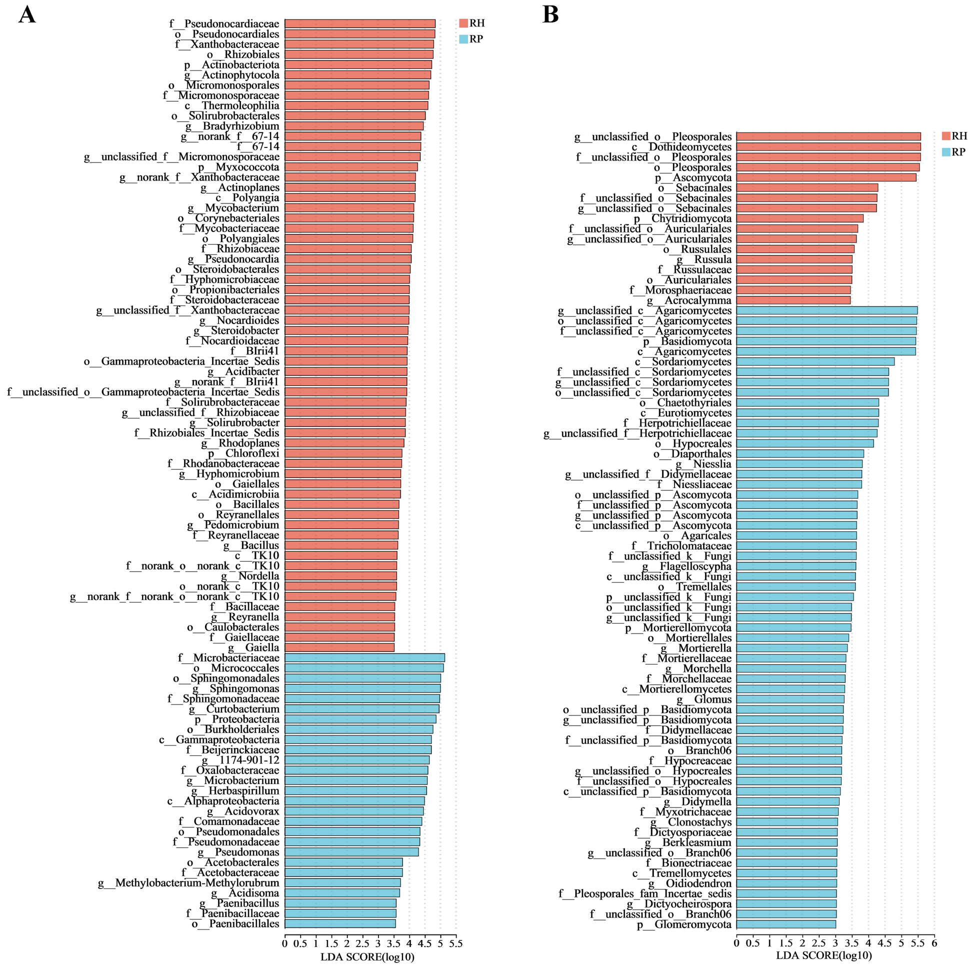

Figure S2. The Linear Discriminant Analysis (LDA) of the root endophytic microbiome of the Verticillium wilt-affected and healthy *C. coggygria*. (A) bacterial community; (B) fungal community. RP represents the Verticillium wilt-affected roots; RH represents healthy roots.

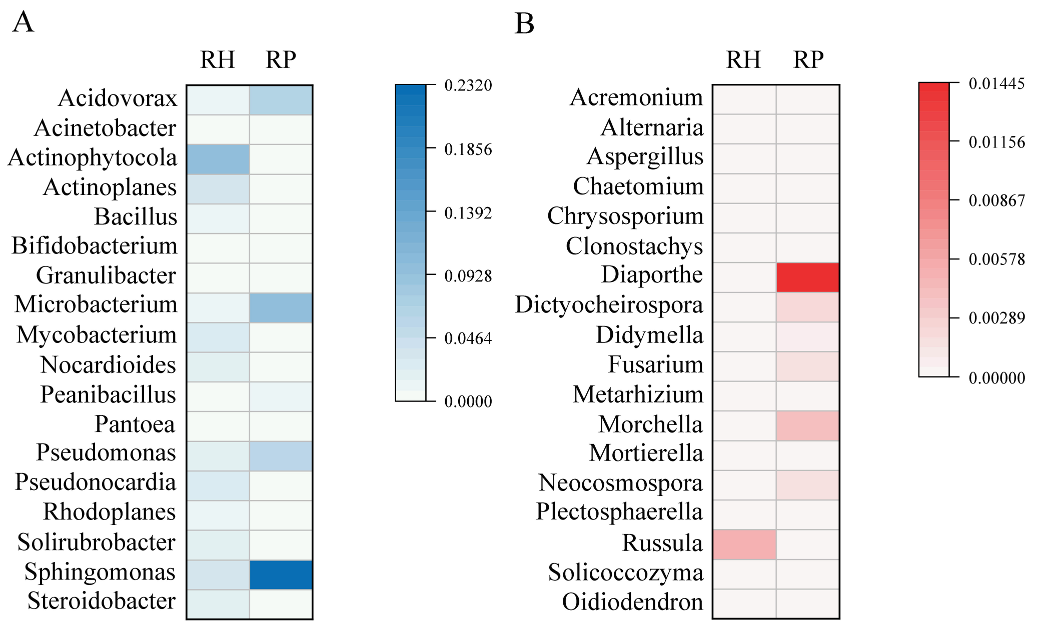

Figure S3. Relative abundance heatmap of the dominant microbial genus of the root endophytic microbiome in Verticillium wilt-affected and healthy *C. coggygria*. (A) bacterial community; (B) fungal community. RP represents the Verticillium wilt-affected roots; RH represents healthy roots.

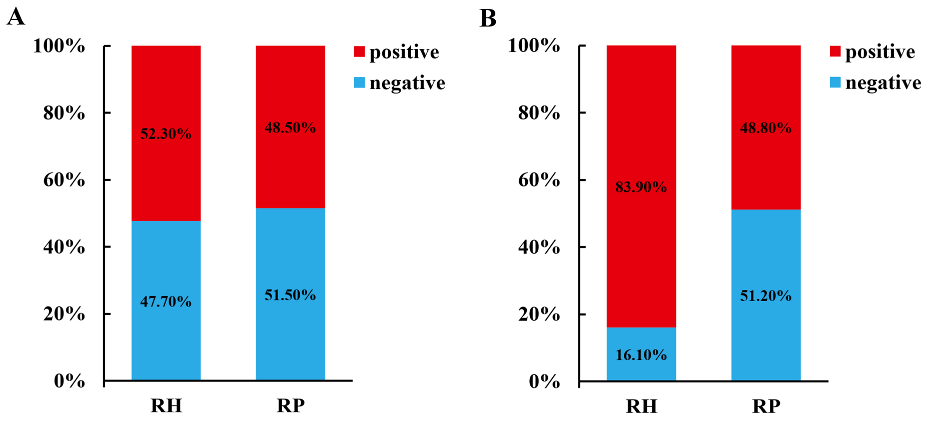

Figure S4. The number of positive and negative interactions in the co-occurrence network of the community in Verticillium wilt-affected and healthy *C. coggygria*. (A) bacterial community; (B) fungal community. RP represents the Verticillium wilt-affected roots; RH represents healthy roots.
